# Symptoms and Physical Signs

**Published:** 1893-09

**Authors:** 


*Symptoms and Physical Signs.* By WILLIAM EWART, M.D. Pp. xiv., 82. London: BAILLIERE, TINDALL & COX. 1892.

This book contains, first, a scheme or syllabus to show the proper method for the systematic clinical investigation of disease, and, secondly, under the heading of the various organs, an index of the chief symptoms and signs met with in different diseases. The index also gives a brief account of some important symptoms, of the alterations of the excretions in disease, and an explanation of the scientific terms used. We think that this index might be made clearer with advantage by being arranged more in dictionary form. The directions for case-taking are good, and students will doubtless be grateful to the author for the help this little book will give them in learning to do their clinical work intelligently, thoroughly, and systematically without waste of time.

